# An Instance of Infirmary Development and Progress

**Published:** 1913-06-14

**Authors:** 


### THE CARE OF THE SICK POOR.

### An Instance of Infirmary Development and Progress.

After thirty-four years' service, Dr. J. Breward Neal is retiring from the position of medical superintendent of the St. John's Hill Infirmary at Wandsworth. Dr. Neal was appointed at a time when the official, if not the public, conscience of the nation was being roused to the iniquitous state of things which once prevailed in Poor-Law institutions. Inspectors from the Local Government Board, notably Dr. Bridges, and such social reformers as Miss Louisa Twining, were insisting that the old order must go. From Dr. Neal one learns that the Guardians of the Wandsworth Union early began to make a move. They were among the first to avail themselves of the Metropolitan Poor Act of 1867, and to build an infirmary which structurally was distinct from the workhouse, and to place it beyond the paramount authority of the workhouse master. The buildings at St. John's Hill, however, were not erected for infirmary purposes alone. They were designed for all the purposes of Poor-Law administration in a Union whose population numbered 200,000, and they were believed to be greatly in advance of their time. In many respects the infirmary block was an improvement on that which it displaced. Dr. Neal, coming to it from asylum work in Cornwall, found it ill-designed, miserably equipped, and in a disgraceful condition of dirt and neglect. It was conducted without organisation or method. There, as in almost every other Poor-Law infirmary, a trained staff of nurses was undreamed of. The infirmary was an institution for "paupers." In England, as in the East, to quote R. F. Burton, the "pauper" was a scoundrel, in respect to whom one felt with Seneca that "pity is a vice." Dr. Neal took charge of a rate-maintained institution at Wandsworth which was probably rather better than such places usually were throughout the country. Within it there was no semblance of discipline or order. Drunkenness, gluttony, and debauch prevailed among the hireling attendants and the able-bodied paupers who ministered to them. The sick were robbed of their nourishment, which was carried outside and sold. Wines and spirits were filched, and were stored up in the hot-water bottles until there was sufficient for a carousal. Men and women were sometimes found in a condition of drunken insensibility. The relatives of the patients were pestered for fees as a reward for services which were never rendered. For the sick the infirmary was

"A lazar-house . . . wherein were laid
Numbers of all diseased—all maladies
Of ghastly spasm or racking torture . . .
Despair tended the sick, busiest from couch to couch;
And over them triumphant Death his dart shook."

There was constant danger of infection. Aseptic or antiseptic treatment was unheard of. In the whole institution there was not a single clinical thermometer nor an instrument to determine the heat of a bath. The windows had not been cleaned for months. The waste would now be thought incredible. The contractor who bought the hogwash sent in a complaint, alleging that this substance was lacking in body and nutriment. It was found on investigation that the inmate helpers had been in the habit of feeding on toast and porter and throwing their meat and vegetables into the waste vat. When the supply of porter was stopped the contractor's hogs grew lean. Dr. Neal set himself from the first to institute a service of trained nurses and to abolish alcoholic stimulants. He had to combat some opposition from members of the Board of Guardians and much prejudice from outside. Professionally, he was almost single-handed, having but one qualified assistant, much of whose time was occupied in dispensing. The paid nurses, so-called, were ordinary domestic servants, whose qualifications were what one would expect from kitchen-maids or charwomen. It was fortunate that Dr. Neal had the hearty support of the majority of the Guardians, at the head of whom was Canon Erskine Clarke, vicar of Battersea. Canon Clarke's influence overshadowed all prejudice, and his determination wore down the opposition of those whose contention was that the "pauper" whether sick or well, should not be pampered at the expense of the thrifty. Within the infirmary Dr. Neal established a school for nurses. Young women were thoroughly taught and trained. The drink problem was tactfully dealt with. The use as well as the abuse of malt liquors was abolished, and in process of time the bill for wines and spirits was brought down from nearly £500 to less than 30s. a year.

In recent years infirmary work has vastly increased, and has broadened. The pauper taint has been removed from the infirmary patient, notwithstanding that some years ago the Wandsworth Guardians, alarmed at the steadily increasing number of cases, caused it to be notified that in law the infirmary is but "the sick wards of the workhouse." Practically a Poor-Law infirmary is a rate-maintained hospital doing hospital work. Years ago the whole of the Union buildings at St. John's Hill became utterly inadequate for the infirmary, in spite of rearrangement and additions. The building of a new institution was nevertheless steadily opposed in certain quarters, until Mr. John Burns, as President of the Local Government Board, sent his chief secretary to Wandsworth on a midnight visit of inspection. He found the floors covered with beds. Patients were lying in corridors and under tables. The overcrowding was appalling. The commencement of a new infirmary could be delayed no longer. Dr. Neal points out that the new institution which has been built at Wandsworth Common strikingly demonstrates the change in ideas with regard to the treatment of the sick poor. The most wealthy hospital in the land is not better equipped than the new Poor-Law infirmary at Wandsworth, and the meanest patient has the benefit of treatment which a decade ago would not have been procurable for a prince.

One institution at St. John's Hill Dr. Neal refers to with pride. It is the nurses' home at the back of the infirmary—comfortable, commodious, and thoroughly homelike. Before the home was built the nursing staff had to make shift with quarters in what were formerly the casual wards. They might have fared worse, for in the board-room one used to hear suggestions of "utilising" a structure—a sort of shed—which was originally put up as an isolation ward. There, in the days when smallpox stalked at noonday through the land, cases of that loathsome disease have developed, and from thence patients have been conveyed in the Guardians' ambulance to the institutions of the Metropolitan Asylums Board. The Guardians had but one ambulance in those days. One's faith in the potency of disinfectants, seeing that an ambulance cannot be vaccinated, is strengthened by the fact that the old infirmary always escaped infection.

Professionally Dr. Neal regards the increase of insanity as the most striking fact in his experience. He cannot attribute this increase to alcoholism, for as often as not the afflicted are total abstainers or extremely moderate drinkers. Perhaps the cause is to be found in the stress of city life, or is traceable to vices which, though not peculiar to cities, are most prevalent there. The frequency of cases of general paralysis of the insane is remarkable, and is far greater in London than in the country.

The magnitude of infirmary work in the Wandsworth Union at the present time is shown by Dr. Maccormac's annual report, which was submitted to the Guardians a few week's ago. During the year there had been 3,307 admissions to the new infirmary. The average daily number of patients was 426. Five hundred and thirty-two operations were performed. The mortality was 11.9 per cent. The work of the nursing staff is exacting, the proportion of nurses to patients being but 1 to 6, as against 1 to 2.5 in a general hospital. The infirmary has an *x*-ray and an electrical department and a pathological laboratory.

There is one unsatisfactory note in the report. Dr. Maccormac, the medical superintendent of St. James' Infirmary at Wandsworth Common—the new infirmary—writes: "I regret to say there has been a heavy sick rate among our nurses. Forty-two nurses were off duty for 715 days, an average of 17 days each. This is largely due to the exacting nature of the work, which frequently obliges the matron to keep the nurses on duty all day."

